# Fusion tags for protein solubility, purification and immunogenicity in *Escherichia coli*: the novel Fh8 system

**DOI:** 10.3389/fmicb.2014.00063

**Published:** 2014-02-19

**Authors:** Sofia Costa, André Almeida, António Castro, Lucília Domingues

**Affiliations:** ^1^Institute for Biotechnology and Bioengineering, Centre of Biological Engineering, University of MinhoBraga, Portugal; ^2^Instituto Nacional de Saúde Dr. Ricardo JorgePorto, Portugal; ^3^Hitag Biotechnology, Lad., Biocant, Parque Technologico de CantanhedeCantanhede, Portugal

**Keywords:** *Escherichia coli*, fusion tags, soluble production, protein purification, tag removal, Fh8 tag, H tag, protein immunogenicity

## Abstract

Proteins are now widely produced in diverse microbial cell factories. The *Escherichia coli* is still the dominant host for recombinant protein production but, as a bacterial cell, it also has its issues: the aggregation of foreign proteins into insoluble inclusion bodies is perhaps the main limiting factor of the* E. coli* expression system. Conversely, *E. coli* benefits of cost, ease of use and scale make it essential to design new approaches directed for improved recombinant protein production in this host cell. With the aid of genetic and protein engineering novel tailored-made strategies can be designed to suit user or process requirements. Gene fusion technology has been widely used for the improvement of soluble protein production and/or purification in *E. coli*, and for increasing peptide’s immunogenicity as well. New fusion partners are constantly emerging and complementing the traditional solutions, as for instance, the Fh8 fusion tag that has been recently studied and ranked among the best solubility enhancer partners. In this review, we provide an overview of current strategies to improve recombinant protein production in *E. coli*, including the key factors for successful protein production, highlighting soluble protein production, and a comprehensive summary of the latest available and traditionally used gene fusion technologies. A special emphasis is given to the recently discovered Fh8 fusion system that can be used for soluble protein production, purification, and immunogenicity in *E. coli*. The number of existing fusion tags will probably increase in the next few years, and efforts should be taken to better understand how fusion tags act in *E. coli*. This knowledge will undoubtedly drive the development of new tailored-made tools for protein production in this bacterial system.

## OUTLINE

Proteins are key elements of life, constituting the major part of the living cell. They play important roles in a variety of cell processes, including cell signaling, immune responses, cell adhesion, and the cell cycle, and their failure is consequently correlated with several diseases.

With the introduction of the DNA recombinant technology in the 1970s, proteins started to be expressed in several host organisms resulting in a faster and easier process compared to their natural sources (Demain and Vaishnav, 2009). *Escherichia coli *remains the dominant host for producing recombinant proteins, owing to its advantageous fast and inexpensive, and high yield protein production, together with the well-characterized genetics and variety of available molecular tools (Demain and Vaishnav, 2009).

The recombinant protein production in *E. coli* has greatly contributed for several structural studies; for instance, about 90% of the structures available in the Protein Data Bank were determined on proteins produced in *E. coli*. (Nettleship et al., 2010; Bird, 2011). The *E. coli* recombinant production has also boosted the biopharmaceutical industry: 30% of the recombinant biopharmaceuticals licensed up to 2011 by the U.S. Food and Drug Administration (FDA) and European Medicines Agency (EMEA) were obtained using this host cell (Ferrer-Miralles et al., 2009; Walsh, 2010; Berlec and Strukelj, 2013).

*Escherichia coli* recombinant protein-based products can also be found in major sectors of the enzyme industry and the agricultural industry with applications ranging from catalysis (e.g., washing detergents) and therapeutic use (e.g., vaccine development) to functional analysis and structure determination (e.g., crystallography; Demain and Vaishnav, 2009).

As a bacterial system, the *E. coli* has, however, limitations at expressing more complex proteins due to the lack of sophisticated machinery to perform posttranslational modifications, resulting in poor solubility of the protein of interest that are produced as inclusion bodies (Demain and Vaishnav, 2009; Kamionka, 2011). Previous studies (Bussow et al., 2005; Pacheco et al., 2012) reported that up to 75% of human proteins are successfully expressed in *E. coli* but only 25% are produced in an active soluble form using this host system. Other problems found within this host system include proper formation of disulfide bonds, absence of chaperones for the correct folding, and the miss-match between the codon usage of the host cell and the protein of interest (Terpe, 2006; Demain and Vaishnav, 2009; Pacheco et al., 2012). Moreover, the industrial culture of *E. coli* leads cells to grow in harsh conditions, resulting in cell physiology deterioration (Chou, 2007; Pacheco et al., 2012).

Despite the above-mentioned issues of *E. coli* recombinant protein production, the benefits of cost and ease of use and scale make it essential to design new strategies directed for recombinant soluble protein production in this host cell. Several strategies have been made for efficient production of proteins in *E. coli*, namely, the use of different mutated host strains, co-production of chaperones and foldases, lowering cultivation temperatures, and addition of a fusion partner (Terpe, 2006; Demain and Vaishnav, 2009). The combination of some of these strategies has improved the soluble production of recombinant proteins in *E. coli*, but the prediction of robust soluble protein production processes is still a “a challenge and a necessity” (Jana and Deb, 2005).

Nowadays, with the aid of genetic and protein engineering, novel tailor-made strategies can be designed to suit user or process requirements.

This review describes the key solubility factors that correlate with successful protein production in *E. coli*, and it presents a comprehensive summary of the available fusion partners for protein production and purification in the bacterial host. A main focus is given to the novel Fh8 fusion system (Hitag®) for soluble protein production, purification and immunogenicity in *E. coli* (Costa, 2013).

## SOLUBLE PROTEIN PRODUCTION IN *ESCHERICHIA COLI*

The production of recombinant proteins requires a successful correlation between the gene’s expression, protein solubility, and its purification (Esposito and Chatterjee, 2006). The production levels of recombinant proteins synthesized in *E. coli* are no longer pointed as a limitation for the success of the overall process, but care should be taken with the protein solubility, which is still a major bottleneck in the field. The downstream processing is deeply associated with an efficient protein production strategy, and thus it must be tailor-designed to maximize the recovery of pure recombinant proteins.

**FIGURE 1:**
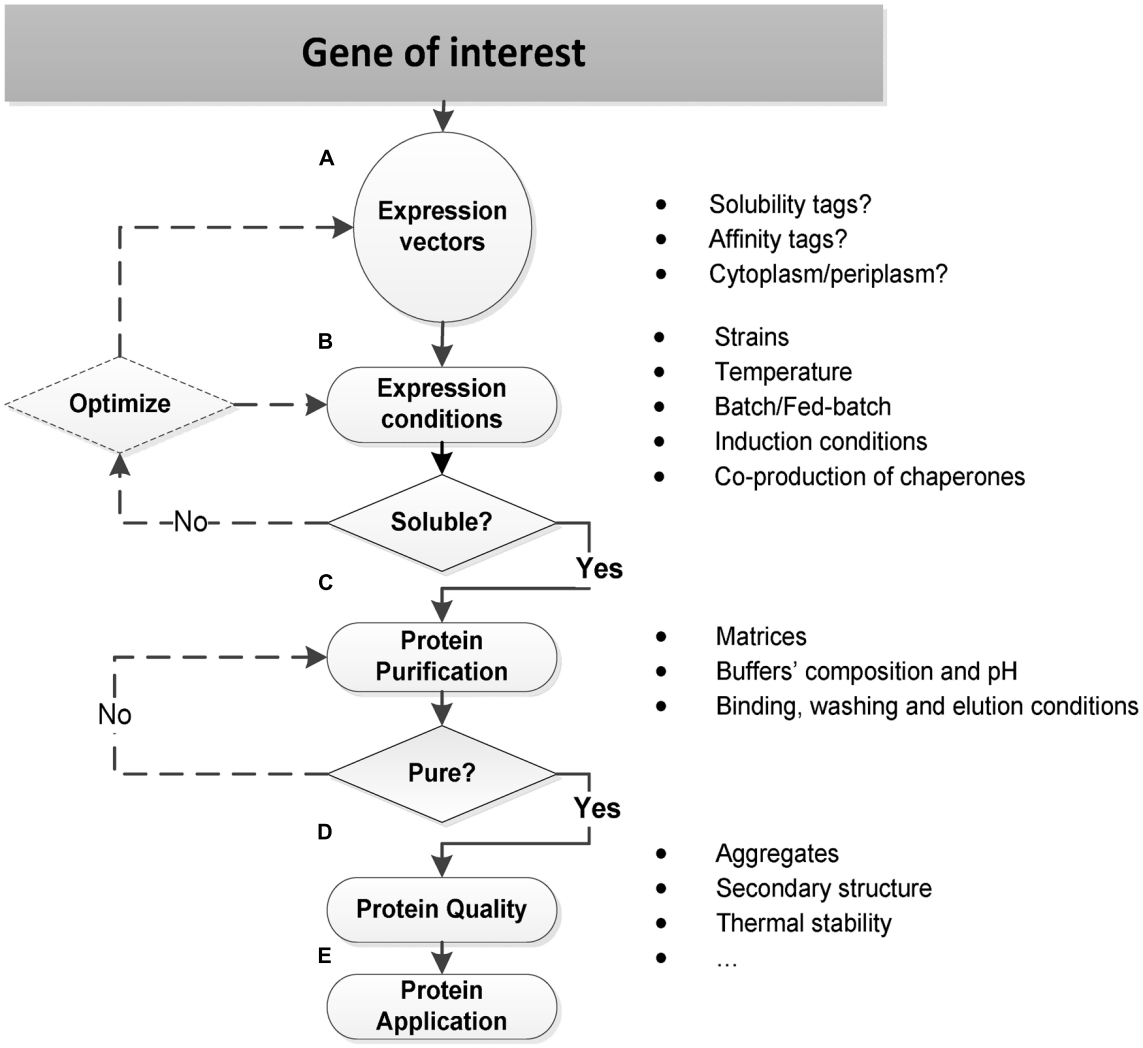
**Strategies for soluble protein production in *E. coli*. (A)** Expression vectors should be carefully selected in order to incorporate specific features that affect the protein production in *E. coli* such as solubility and/or affinity fusion tags, and to direct the protein synthesis to the *E. coli* cytoplasm or periplasm. Other important features include: the replicon, antibiotic-resistance markers, and transcriptional promoters (Jana and Deb, 2005; Sorensen and Mortensen, 2005a). **(B)** The optimization of expression conditions often directs the soluble protein production in *E. coli*, and it relies on a trial-and-error basis: to get a soluble TP, it may require the selection and testing of several engineered *E. coli* strains and cultivation conditions, and sometimes the initial expression vector has also to be re-designed. **(C)** The protein purification strategy should already be defined at the beginning when selecting the expression vector: if an affinity tag is incorporated, then a first affinity chromatography step should be conducted. On the other hand, if an affinity tag is prohibit, other strategies, namely, ion exchange, size exclusion, or hydrophobic interaction chromatography should be tested. After the first purification step, the TP may or may not be sufficiently pure. When it is not pure, further purification steps with other chromatographic strategies need to be conducted. **(D–E)** The protein quality is an essential requirement for many structural and functional application studies: a purified soluble protein may be aggregated, without a defined secondary structure, and it may also present a low thermal stability. Therefore, a biophysical characterization is often required before proceeding to the final protein’s application.

All these three properties – expression, solubility, and purification – shall always be considered together as determinants for the effective protein production in *E. coli*. Several aspects are though essential for each individual success, as resumed in **Figure 1** and described.

### STRATEGIES FOR THE SUCCESSFUL AND EFFICIENT SOLUBLE PROTEIN PRODUCTION IN *E. COLI* – PREVENTION OF PROTEIN AGGREGATION

*Escherichia coli* recombinant protein production systems are designed to achieve a high accumulation of soluble protein product in the bacterial cell. However, a strong and rapid protein production can lead to stressful situations for the host cell, resulting in protein misfolding *in vivo*, and consequent aggregation into inclusion bodies (Schumann and Ferreira, 2004; Sorensen and Mortensen, 2005a,b; Sevastsyanovich et al., 2010). For instance, macromolecular crowding of proteins at high concentrations in the *E. coli* cytoplasm often impairs the correct folding of proteins, leading to the formation of folding intermediates that, when inefficiently processed by molecular chaperones, promote inclusion body formation (Sorensen and Mortensen, 2005a,b).

Strategies that direct the soluble production of proteins in *E. coli* are, thus, envisaged, and become more attractive than protein refolding procedures from inclusion bodies.

Several methods have been shown to prevent or decrease protein aggregation during protein production in *E. coli* on a trial-and-error basis, including:

(i) *Lower expression temperatures*: bacteria cultivation at reduced temperatures is often used to reduce protein aggregation, since it slows down the rate of protein synthesis and folding kinetics, decreasing the hydrophobic interactions that are involved in protein self-aggregation (Schumann and Ferreira, 2004; Sorensen and Mortensen, 2005b). Low cultivation temperatures can also reduce or impair protein degradation due to a poor activity of heat shock proteases that are usually induced during protein overproduction in *E. coli* (Chesshyre and Hipkiss, 1989). This strategy has, however, some drawbacks as the reduction of temperature can also affect replication, transcription, and translation rates, besides decreasing the bacterial growth and protein production yields. Nevertheless, these limitations can be circumvented by the use of cold-inducible promoters that maximize protein production under low temperature conditions (Mujacic et al., 1999).

(ii) *E. coli*-engineered host strains: *E. coli* mutant strains are a significant advance toward the soluble production of difficult recombinant proteins. Several targeted strain strategies have been developed through the introduction of DNA mutations that affect protein synthesis, degradation, secretion, or folding (reviewed in Makino et al., 2011), including: engineered strains for improved protein processing at low temperatures, such as the Arctic Express strain (Agilent Technologies); mutated strains that increase mRNA stability by attenuation of RNases activity, which is responsible for the shorter half-life of mRNA in *E. coli* cells (Lopez et al., 1999); engineered strains that supply extra copies of rare tRNAs, such as the Rosetta strains (Invitrogen) and the BL21 Codon Plus strains (Novagen; Baca and Hol, 2000; Sorensen et al., 2003b); mutant strains that facilitate disulfide bond formation and protein folding in the *E. coli* cytoplasm by render it oxidizing due to mutations in glutathione reductase (*gor*) and thioredoxin reductase (*trxB*) genes, and/or by co-production of Dsb proteins (Bessette et al., 1999; Lobstein et al., 2012), such as the Origami strains (Novagen) or the new SHuffle strain (New England Biolabs; Lobstein et al., 2012); and C41 and C43 (Avidis) BL21 (DE3) mutant strains that improve the synthesis of membrane proteins (Miroux and Walker, 1996).

(iii) *Cultivation conditions*: protein production can be efficiently improved by the use of high cell-density culture systems like batch, which offers a limited control of the cell growth, and fed-batch, which allows the real time optimization of growth conditions (Sorensen and Mortensen, 2005b). The composition of the cell growth medium and the fermentation variables such as temperature, pH, induction time, and inducer concentration are also essential for the prevention of protein aggregation, whereby a careful optimization improves the yield and quality of soluble protein production (Jana and Deb, 2005).

(iv) *Co-production of molecular chaperones and folding modulators*: the initial folding of proteins can be assisted by molecular chaperones that prevent protein aggregation through binding exposed hydrophobic patches on unfolded, partially folded or misfolded polypeptides, and traffic molecules to their subcellular destination. Protein aggregation is also prevented by folding catalysts that catalyze important events in protein folding such as the disulfide bond formation (Kolaj et al., 2009). A low concentration of these folding modulators in the cell often results in protein folding failures; thereby their co-production together with the target protein becomes a suitable strategy for the improvement of soluble protein production in *E. coli* (reviewed in Thomas et al., 1997; Schlieker et al., 2002; Baneyx and Palumbo, 2003; Hoffmann and Rinas, 2004; Betiku, 2006; Gasser et al., 2008; Kolaj et al., 2009). Chaperones like trigger factor, DnaK, GroEL, members of the heat shock protein Hsp70 and Hsp60 families (hsHsp proteins), and ClpB assist protein folding in the *E. coli* cytoplasm, and their individual or cooperative activities presents different contributions for target protein solubility (Nishihara et al., 1998; Kuczynska-Wisnik et al., 2002; Schlieker et al., 2002; Deuerling et al., 2003; de Marco and De Marco, 2004; de Marco et al., 2007).

(v) *Fusion partner proteins*: in contrast to the above-mentioned strategies, the use of fusion partners involves the target protein engineering. Fusion partners are very stable peptide or protein molecules soluble expressed in *E. coli* that are genetically linked with target proteins to mediate their solubility and purification.

### CHROMATOGRAPHIC STRATEGIES FOR RECOMBINANT PROTEIN PURIFICATION

The protein purification accounts for most of the expenses in recombinant protein production. Hence, the design of a straightforward and cost-effective protein isolation and purification is one of the first steps to be considered in the production strategy.

There is no single or simple way to purify all kinds of proteins because of their diversity and different properties. Therefore, several strategies have been developed in the past decades to address a broad range of samples. With the introduction of recombinant DNA technology in the seventies, novel affinity tagging methodologies have revolutionized protein purification processes and several easy-to-use affinity tags have emerged since then. Besides the isolation of recombinant proteins, the purification process is also used to concentrate the desired protein. The target protein is usually first designed to be affinity tagged, thus facilitating the purification process and allowing the target protein to maintain its properties without interacting directly with a matrix. However, if the target protein cannot be affinity tagged or if further purification is needed, other purification strategies are added to the process.

When designing a purification strategy, one must consider the final goal of the target protein to be purified. For instance, recombinant proteins for therapeutic and biomedical applications require a high-level of protein purity and they probably should undergo several subsequent purification steps.

The available protein purification methodologies separate the target proteins according to differences between the properties of the protein to be purified and properties of the rest of the protein mixture. Recombinant proteins are nowadays purified using column chromatography in scales from micrograms or milligrams in research laboratories to kilograms in industrial settings. The purification of a target protein from a crude cell extract is, however, not always easy and even with all the progresses achieved so far, additional physicochemical-based chromatography methods such as size exclusion (SEC), ion exchange (IEX), and hydrophobic interaction (HIC) are often used to complement the affinity tagging. These methods rely on minor differences between various proteins properties such as size, charge, and hydrophobicity, respectively (GE Healthcare, 2010).

In a traditional purification pipeline, the chromatography starts with a capturing step, where the target protein binds to the absorbent while the impurities do not. Then, weakly bound proteins are washed out of the column, and conditions are changed so that the target protein is eluted from the column.

#### Size exclusion chromatography

This technique is a non-binding method that separates protein samples with different molecular sizes under mild conditions. Size exclusion chromatography (SEC) can be used for protein purification, in which it usually dilutes the sample, or for group separation, which is mainly used for desalting and buffer exchange of samples. This technique is ideal for the final polishing in a multiple-step purification strategy. Analytical SEC allows the determination of the hydrodynamic radius of protein molecules and the corresponding molecular weight (GE Healthcare, 2010).

#### Ion exchange chromatography

This technique separates proteins with different surface charge and it offers a high-resolution separation combined with high sample loading capacity. The purification relies on a reversible interaction between a charged protein and an oppositely charged chromatography medium. Proteins purified by ion exchange chromatography (IEX) are usually obtained in a concentrated form. The net surface charge of proteins is influenced by the surrounding pH: when the pH is above the protein isoelectric point (pI), the target protein has a negatively charged shield that is used for binding to a positively charged anion exchanger; when the pH is below its pI, the target protein has a positively charged shield that is used for binding to a negatively charged cation exchanger. The IEX purification protocol is initiated under low ionic strength, and the conditions are then changed so that the bound substances can be eluted differentially by increasing salt concentration or changing pH using a gradient or stepwise strategy. In general, the IEX is used to bind the target protein, but it can also be used to bind impurities when required. The IEX is the most common technique used for the capture step in a multiple-step purification strategy, but it can be used in the intermediate step as well (GE Healthcare, 2010).

#### Hydrophobic interaction chromatography

Hydrophobic interaction chromatography (HIC) separates proteins according to differences in their surface hydrophobicity by using a reversible interaction between non-polar regions on the surface of these proteins and the immobilized hydrophobic ligands of a HIC medium (Queiroz et al., 2001). The proteins are separated according to differences in the amount of exposed hydrophobic amino acids. This technique is ideal for capture and intermediate steps in a multiple-step purification strategy.

The interaction between hydrophobic proteins and a HIC medium is influenced significantly by several parameters (reviewed in Queiroz et al., 2001; Lienqueo et al., 2007), including:

(i) *The type of the ligand and degree of substitution*: the type of immobilized ligand (alkyl or aryl) determines the protein adsorption selectivity of the HIC adsorbent. In general, alkyl ligands show more pure hydrophobic character than aryl ligands. The protein binding capacities of HIC adsorbents increase with increased degree of substitution of immobilized ligand. The degree of substitution is the average number of substituent groups attached per milliliter of gel, and it correlates with the protein binding capacities of HIC adsorbents as follows: higher binding capacities are obtained with an increased degree of substitution of immobilized ligand. At a reasonably high degree of ligand substitution, the apparent binding capacity of the adsorbent remains constant (the plateau is reached) but the strength of the interaction increases. Solutes bound under such circumstances are difficult to elute due to multi-point attachment (GE Healthcare, 2006).

(ii) *The type of base matrix*: the matrix should be neutral to avoid ionic interactions between the protein and the matrix, and it should also be hydrophilic. The two most widely used matrices are strongly hydrophilic carbohydrates, such as cross-linked agarose, or synthetic copolymer materials (GE Healthcare, 2006).

(iii) *The type and concentration of salt*: a high salt concentration enhances the interaction, while lowering the salt concentration weakens the interaction. The effect of the salt type on protein retention follows the Hofmeister series for the precipitation of proteins from aqueous solutions (Collins and Washabaugh, 1985; Zhang and Cremer, 2006). In Hofmeister series, the chaotropic salts (magnesium sulfate and magnesium chloride) randomize the structure of the liquid water and thus tend to decrease the strength of hydrophobic interactions. In contrast, the kosmotropic salts (sodium, potassium, or ammonium sulfates) promote hydrophobic interactions and protein precipitation, due to higher “salting-out” or molar surface tension increment effects.

(iv) *pH*: when pH is close to a protein’s pI, net charge is zero and hydrophobic interactions are maximum, due to the minimum electrostatic repulsion between the protein molecules allowing them to get closer. In general, an increase in the pH weakens the hydrophobic interaction probably due to an increased titration of charged groups, thereby leading to an increase of protein hydrophilicity. A decrease of the pH may result in an increase of hydrophobic interactions. However, the effect of pH in HIC is not always straightforward (GE Healthcare, 2006).

(v) *Temperature*: the role of temperature in HIC is complex, but in general, increased temperatures enhance the protein retention. Careful should be taken when conducting protein purifications at room temperature as the protein performance in the HIC will probably not be reproducible in a cold room, and vice versa.

(vi) *Additives*: low concentrations of water-miscible alcohols, detergents, and aqueous solutions of chaotropic (“salting-in”) salts result in a weakening of the protein–ligand interactions in HIC leading to the desorption of the bound solutes. The non-polar parts of alcohols and detergents compete with the bound proteins for the adsorption sites on the HIC media resulting in the displacement of the latter. Chaotropic salts affect the ordered structure of water and/or that of the bound proteins. Both types of additives also decrease the surface tension of water thus weakening the hydrophobic interactions to give a subsequent dissociation of the ligand–solute complex. The use of additives should be carefully considered as they might compromise the target protein structure and activity (GE Healthcare, 2006, 2010).

Proteins bound to HIC media can be eluted using some of the above-mentioned conditions such as reduced salt concentration, increased pH, or addition of alcohols or detergents (Lienqueo et al., 2007), but trial-and-error experiments should be conducted to select the best option for each specific target protein.

Besides protein purification, the HIC methodology offers several potentialities in protein production, being described as one of the most used strategies for endotoxin clearance (Wilson et al., 2001; Magalhães et al., 2007; Ongkudon et al., 2012). It can also be used for protein refolding (Hwang et al., 2010).

The HIC methodology has been applied for the purification of calcium-binding proteins (CaBPs; Rozanas, 1998; Shimizu et al., 2003; McCluskey et al., 2007). These proteins expose a large hydrophobic surface in the presence of calcium that can absorb to hydrophobic matrices such as phenyl sepharose, even in the presence of low salt concentration. Most of the contaminant proteins will not bind under these conditions, which benefits the recovery of a pure CaBP. The elution step is often achieved by removal of the bound calcium through the use of chelating agents like EDTA (Rozanas, 1998).

#### Affinity chromatography

This technique separates proteins through a reversible interaction between the target protein and a specific ligand attached to a chromatographic matrix. The interaction can be performed via an antibody (biospecific interaction), or via an immobilized metal ion (non-biospecific interaction) or dye substance. The affinity chromatography usually offers high selectivity and resolution together with an intermediate-high capacity. The sample is first bound to the ligand using favorable conditions for that binding. Then, the unbound material is washed out of the column and the elution of pure protein is achieved using a competitive ligand or by changing the pH, ionic strength or polarity (GE Healthcare, 2010). This purification strategy can profit from the use of recombinant DNA technology as the affinity tag can be fused to the protein of interest during cloning and it is further presented in the next section.

### FUSION PROTEIN TECHNOLOGY

Fusion partners or tags are used in *E. coli* to improve protein production yields, solubility and folding, and to facilitate protein purification. They can also confer specific properties for target proteins characterization and study, such as protein immunodetection, quantification, and structural and interactional studies (Malhotra, 2009). Fusion partners can also be of use when producing toxic proteins. An example is the production of antimicrobial peptides (AMPs) by *E. coli* using cellulose binding modules as fusion partner (Guerreiro et al., 2008; Ramos et al., 2010, 2013). The use of carbohydrate-binding modules (CBMs) as fusion partner has also been applied for targeting peptides and/or functionalizing specific supports/biomaterials for biomedical applications (Moreira et al., 2008; Andrade et al., 2010a,b; Pértile et al., 2012). Besides the fusion(s) partner(s) coding gene, *E. coli* expression vectors can contain a protease recognition sequence between the fusion partner coding gene and the passenger protein coding gene that allows the tag removal when the latter protein is for using in protein therapies, vaccine development and structural analyses.

Some fusion partners also protect target proteins from degradation by promoting the translocation of the passenger protein to different cellular locations, where less protease content exists (Butt et al., 2005). Both maltose-binding protein (MBP) and small ubiquitin related modifier (SUMO) fusion partners present this feature, passing target proteins from the *E. coli* cytosol for cell membrane and nucleus, respectively (Nikaido, 1994; Kishi et al., 2003).

When designing a fusion strategy, the choice of the fusion partner depends on several aspects (Young et al., 2012), including:

(i) *Purpose of the fusion*: is it for solubility improvement or for affinity purification? Nowadays, a variety of fusion tags that render different purposes are available, and systems containing both solubility and affinity tags like, for instance, the dual hexahistine (His_6_)-MBP tag, can be designed in order to get a rapid “in one step” protein production. Some protein tags can also function in both affinity and solubility roles, as for instance, the MBP or glutathione-*S*-transferase (GST; Esposito and Chatterjee, 2006). If the fusion tag is to be used in protein purification, the cost and buffer conditions are often the criteria for selection. For instance, proteins that require chelating agents as EDTA are not suitable for immobilized metal affinity chromatography (IMAC) via the His_6_ tag as nickel ions in the affinity matrix are chelated by EDTA (Malhotra, 2009).

(ii) *Amino acid composition and size*: these two factors should be considered when selecting a fusion partner because target proteins may require larger or smaller tags depending on their application. Larger tags can present a major diversity in the amino acid content, and will impose a metabolic burden in the host cell different from that imposed by small tags (Malhotra, 2009).

(iii) *Required production levels*: structural studies require higher protein production levels that can be rapidly achieved with a larger fusion tag, which has strong translational initiation signals, whereas the study of physiological interactions demands for lower production levels and small tags (Malhotra, 2009).

(iv) *Tag location*: fusion partners can promote different effects when located at the N-terminus or C-terminus of the passenger protein. Usually, N-terminal tags are advantageous over C-terminal tags because: (1) they provide a reliable context for efficient translation initiation, in which fusion proteins take advantage of efficient translation initiation sites on the tag; (2) they can be removed leaving none or few additional residues at the native N-terminal sequence of the target protein, since most of endoproteases cleave at or near the C-terminus of their recognition sites (Waugh, 2005; Malhotra, 2009).

Fusion tags can be incorporated using different strategies: affinity and solubility tags are set individually or together, and sites for protease cleavage are designed between the fusion tags and target proteins.

#### Solubility enhancer partners

In spite of all the approaches conducted so far, the choice of a fusion partner is still a trial-and-error experience. Fusion partners do not perform equally with all target proteins, and each target protein can be differentially affected by several fusion tags (Esposito and Chatterjee, 2006). In the past decade, parallel high throughput (HTP) screenings using different fusion partners have developed soluble protein production, and facilitated a rapid, tailored, and cost-effective choice of the best fusion partner for each target protein (Hammarstrom et al., 2002; Shih et al., 2002; Dyson et al., 2004; Dummler et al., 2005; Cabrita et al., 2006; Hammarstrom, 2006; Marblestone et al., 2006; Kim and Lee, 2008; Kohl et al., 2008; Ohana et al., 2009; Bird, 2011).

The mechanisms by which fusion tags enhance the solubility of their partner proteins remain unclear, but several hypotheses have been suggested (Butt et al., 2005; Nallamsetty and Waugh, 2007):

(i) Fusion proteins form *micelle-like structures*: misfolded or unfolded proteins are sequestered and protected from the solvent and the soluble protein domains face outward;

(ii) Fusion partners *attract chaperones*: the fusion tag drives its partner protein into a chaperone-mediated folding pathway. MBP and N-utilization substance (NusA) are two fusion tags that present this mechanism, being previously reported to interact with GroEL in *E. coli* (Huang and Chuang, 1999; Douette et al., 2005);

(iii) Fusion partners have an *intrinsic chaperone-like activity*: hydrophobic patches of the fusion tag interact with partially folded passenger proteins, preventing their self-aggregation, and promoting their proper folding. MBP was previously reported to act also as a chaperone in the fusion context (Kapust and Waugh, 1999; Fox et al., 2001). Solubility enhancer partners may thus play a passive role in the folding of their target proteins, reducing the chances for protein aggregation (Waugh, 2005; Nallamsetty and Waugh, 2006);

(iv) Fusion partners *net charges*: highly acidic fusion partners were suggested to inhibit protein aggregation by electrostatic repulsion (Zhang et al., 2004; Su et al., 2007).

A large variety of solubility enhancer tags are available (**Table 1**), including the well-known MBP, NusA, thioredoxin (TrxA), GST, and SUMO, and several other novel moieties recently discovered, for instance, the Fh8 tag.

**Table 1:** Solubility enhancer tags [adapted from Esposito and Chatterjee (2006), Malhotra (2009)].

Tag	Protein	Size (aa)	Organism	Reference
Fh8	*Fasciola hepatica* 8-kDa antigen	69	*F. hepatica*	Costa (2013), Costa et al. (2013a,b)
MBP	Maltose-binding protein	396	*Escherichia coli*	di Guan et al. (1988), Kapust and Waugh (1999)
NusA	N-utilization substance	495	*E. coli*	Davis et al. (1999)
Trx	Thioredoxin	109	*E. coli*	Lavallie et al. (1993)
SUMO	Small ubiquitin modified	~100	*Homo sapiens*	Butt et al. (2005), Marblestone et al. (2006)
GST	Glutathione-*S*-transferase	211	*Schistosoma japonicum*	Smith and Johnson (1988)
SET	Solubility-enhancer peptide sequences	<20	Synthetic	Zhang et al. (2004)
GB1	IgG domain B1 of Protein G	56	*Streptococcus sp.*	Zhou et al. (2001), Cheng and Patel (2004)
ZZ	IgG repeat domain ZZ of Protein A	116	*Staphylococcus aureus *	Rondahl et al. (1992), Inouye and Sahara (2009)
HaloTag	Mutated dehalogenase	~300	*Rhodococcus sp.*	Ohana et al. (2009)
SNUT	*S*olubility e*N*hancing *U*biquitous *T*ag	147	*Staphylococcus aureus*	Caswell et al. (2010)
Skp	Seventeen kilodalton protein	161	*E. coli*	Esposito and Chatterjee (2006)
T7PK	Phage T7 protein kinase	~240	*Bacteriophage T7*	Esposito and Chatterjee (2006)
EspA	*E. coli* secreted protein A	192	*E. coli*	Cheng et al. (2010)
Mocr	Monomeric bacteriophage T7 0.3 protein (Orc protein)	117	*Bacteriophage T7*	DelProposto et al. (2009)
Ecotin	*E. coli* trypsin inhibitor	162	*E. coli*	Malik et al. (2006, 2007)
CaBP	Calcium-binding protein	134	*Entamoeba histolytica *	Reddi et al. (2002)
ArsC	Stress-responsive arsenate reductase	141	*E. coli*	Song et al. (2011)
IF2-domain I	N-terminal fragment of translation initiation factor IF2	158	*E. coli*	Sorensen et al. (2003a)
Expressivity tag (part of IF2-domain I)	N-terminal fragment of translation initiation factor IF2	7 (21 nt)	*E. coli*	Hansted et al. (2011)
RpoA, SlyD, Tsf, RpoS, PotD, Crr	Stress-responsive proteins	329, 196, 283, 330, 348, 169	*E. coli*	Ahn et al. (2007), Han et al. (2007a,b), (2007c), Park et al. (2008)
msyB, yjgD, rpoD	*E. coli* acidic proteins	124, 138, 613	*E. coli*	Su et al. (2007), Zou et al. (2008)

*aa– amino acids; nt– nucleotides.*

*MBP* is a large (43 kDa) periplasmic and highly soluble protein of* E. coli* that acts as a solubility enhancer tag (Kapust and Waugh, 1999; Fox et al., 2001), and it has a native affinity property to function as a purification handle.

MBP plays an important role in the translocation of maltose and maltodextrins (Nikaido, 1994): it has a natural protein-binding site that it uses to interact with other proteins involved in maltose signaling and chemotaxis, and it has a large hydrophobic cleft close to this site that undergoes conformational changes upon maltose binding (Fox et al., 2001).

When used in the fusion context, MBP promotes target protein solubility by showing chaperone intrinsic activity (Kapust and Waugh, 1999; Bach et al., 2001; Fox et al., 2001), and it is more efficient at the N-terminus of the target proteins rather than at the C-terminus (Sachdev and Chirgwin, 2000). In fact, MBP promotes the proper folding of the target protein by interacting with the latter, and occluding its self-association. This passive role of MBP in protein folding is correlated with the large hydrophobic area exposed on its surface, which is responsible for the contact with other proteins in the maltose transport apparatus (Kapust and Waugh, 1999; Fox et al., 2001). Hence, the MBP hydrophobic cleft is pointed as the site where fused polypeptides interact with the fusion partner (Kapust and Waugh, 1999; Fox et al., 2001; Nallamsetty and Waugh, 2007), similar to what it is reported for GroEL and DnaK molecular chaperones (Buckle et al., 1997; Chatellier et al., 1999; Tanaka and Fersht, 1999). The presence of this cleft can explain why only certain soluble proteins like MBP act as solubilizing agents. Moreover, MBP presents certain conformational flexibility associated with the cleft; thereby it can adjust its shape to accommodate several different polypeptides.

MBP fusion proteins bind to immobilized amylose resins, but this binding is highly dependent on the nature of the passenger protein as it can block or reduce the amylose interaction (Pryor and Leiting, 1997). Difficulties found in the binding of MBP fusion proteins to amylose resins corroborate the hypothesis that target proteins interact with MBP via its binding site (Fox et al., 2001).

Other affinity tags, specific proteases and protein cultivation strategies are being employed together with MBP to improve protein soluble production, purification and native protein recovery, as for instance, His_6_-MBP fusions (Nallamsetty et al., 2005), His_6_-MBP-TEV fusions (Rocco et al., 2008), MBP-His_6_-Smt3 fusions in which the *Saccharomyces cerevisiae* Smt3 protein is used for protein processing by proteolytic cleavage between the MBP-His_6_ tags and the protein of interest (Motejadded and Altenbuchner, 2009), and secretion of MBP fusion protein into the culture medium (Sommer et al., 2009).

Several commercial expression vectors containing the MBP tag are available for cytoplasmic and periplasmic production of target proteins, including the pMAL series (New England Biolabs) and pIVEX (Roche).

*NusA* is a transcription termination/anti-termination protein that promotes/prevents RNA polymerase pausing when acting alone or when included in the anti-termination complex, respectively. NusA (55 kDa) is used as a fusion partner to confer stability and high solubility to its target proteins (De Marco et al., 2004; Dummler et al., 2005; Turner et al., 2005). The NusA ability to improve the soluble production of fusion proteins may be correlated with its intrinsically solubility and biological activity in *E. coli*. NusA slows down translation at the transcriptional pauses, offering more time for protein folding (Davis et al., 1999; De Marco et al., 2004). In contrast to MBP, NusA does not present an intrinsic affinity property, therefore requiring the addition of an affinity tag for efficient protein production, as for instance, the His_6_ tag (Davis et al., 1999). As for MBP, several strategies have been exploited to use the NusA solubility enhancer fusion partner with purification tags and specific proteases like the pETM60 vector (EMBL; De Marco et al., 2004) that render the production of a NusA–His_6_–TEV fusion protein, or the pET43 (Novagen), that offers the same NusA–His_6_ fusion protein but with a thrombin and enterokinase cleavage sites between the fusion tags and target proteins.

In spite of the different physiochemical and structural properties, as well as different biological functions, *MBP* and *NusA* are often reported to promote similar solubility improvements in their target proteins, being ranked as two of the best tags for making soluble proteins (Shih et al., 2002; Kohl et al., 2008; Bird, 2011). Both fusion partners were reported to probably work by similar mechanisms, in which NusA, like MBP, plays a passive role on the target protein folding (Nallamsetty and Waugh, 2006).

*TrxA*, or* Trx*, is a 12-kDa intracellular thermostable protein of *E. coli* that is highly soluble expressed in its cytoplasm (Young et al., 2012). The *E. coli* Trx can be used for co-production with a target protein, improving the solubility of the latter (Yasukawa et al., 1995). Trx is also commonly employed as a fusion tag to avoid inclusion body’s formation in recombinant protein production by taking advantage of its intrinsic oxido-reductase activity responsible for the reduction of disulfide bonds through thio-disulfide exchange (Stewart et al., 1998; LaVallie et al., 2000; Young et al., 2012). The fusion partner Trx can be placed both at the N- or C-terminal of target proteins (LaVallie et al., 2000) but this fusion partner is more effective at the N-terminal of the target protein (Terpe, 2003; Dyson et al., 2004). In some HTP screenings (Hammarstrom et al., 2002; Dyson et al., 2004; Kim and Lee, 2008), the Trx fusion partner improves target protein solubility similar to MBP tag, being considered one of the best choices for protein production in *E. coli*.

Unlike MBP, Trx does not have intrinsic affinity properties, thus requiring an additional fusion tag for protein purification such as the His_6_ tag. The pET32 (Novagen), one of the commercially available vectors for Trx tagging, carries this dual-fusion partners for protein production and purification (Austin, 2003).

Trx fusion partner can also be useful in protein crystallization of certain target proteins because it readily forms several crystals itself, and it offers a rigid connection to the target protein, which is an essential feature for blocking conformational heterogeneity usually found in various attempts of fusion proteins crystallization (Smyth et al., 2003; Corsini et al., 2008).

Small ubiquitin related modifier is a small protein (~11 kDa) found in yeast (one single gene coding for Smt3) and vertebrates (three genes coding for SUMO-1, SUMO-2, and SUMO-3; Kawabe et al., 2000) that has recently been used as an effective N-terminal solubility enhancer fusion partner, offering advantages over other fusion systems (Marblestone et al., 2006; Bird, 2011).

The robust SUMO protease (catalytic domains of Ulp1) offers significant advantages over other endoproteases because it recognizes the tertiary structure of SUMO, and consequently it does not present unspecific cleavage of the protein linear amino acid sequence. Moreover, when used for tag removal, SUMO protease generates a cleaved target protein with its native N-terminal amino acid composition (Malakhov et al., 2004; Marblestone et al., 2006).

Small ubiquitin related modifier promotes the proper folding and solubility of its target proteins possibly by exerting chaperoning effects in a similar mechanism to the described for its structural homolog Ubiquitin (Ub; Khorasanizadeh et al., 1996). Ub was reported to be the nature’s fastest folding protein, and SUMO also presents a tight, rapidly folding soluble structure (Marblestone et al., 2006). In addition, Ub and Ub-like proteins (Ulp) have a highly hydrophobic inner core and a hydrophilic surface that, together with such a rapid folding, may explain the SUMO’s behavior as a nucleation site for the proper folding of target proteins (Malakhov et al., 2004; Marblestone et al., 2006).

Small ubiquitin related modifier fusion proteins or peptides are usually purified by affinity chromatography using the His_6_ tag (Lee et al., 2008; Gao et al., 2010; Wang et al., 2010; Satakarni and Curtis, 2011). Due to its unique features, SUMO technology has being constantly explored, and novel strategies for a facile and rapid protein production are now available, as the SUMO–intein system (Wang et al., 2012). The SUMO fusion partner is also available for recombinant protein production in other host cells, namely, insect cells and other eukaryotic cells (Panavas et al., 2009).

Glutathione-*S*-transferase from *Schistosoma japonicum* (26 kDa) that has been used as an affinity fusion partner for the single-step purification of its target proteins (Smith and Johnson, 1988). GST can also promote protein soluble production in *E. coli*, being more efficient when positioned at the N-terminal rather than at the C-terminal end (Malhotra, 2009). This fusion partner can protect its target protein from the proteolytic degradation, stabilizing it into the soluble fraction (Kaplan et al., 1997; Hu et al., 2008; Young et al., 2012). In spite of performing quite well in some HTP studies (Dummler et al., 2005; Cabrita et al., 2006; Kim and Lee, 2008), GST is often a poor solubility tag when compared to other commonly fusion partners, rendering the target protein production into inclusion bodies (Hammarstrom et al., 2002; Dyson et al., 2004; Hammarstrom, 2006; Kohl et al., 2008; Ohana et al., 2009).

Glutathione transferases are dimeric enzymes that catalyze the nucleophilic addition of the thiol of glutathione to a wide range of hydrophobic electrophilic molecules (Ketterer, 2001). Taking this feature into account, GST can be useful for monitoring the protein production and purification via its catalytic activity, and the purification of GST fusion proteins can be easily performed by affinity chromatography using glutathione derivates immobilized into a solid support (Viljanen et al., 2008). GST fusion proteins can be eluted with glutathione under mild conditions (Vinckier et al., 2011).

A major disadvantage for using GST as solubility and affinity tag relies on its oligomerized form: GST has four solvent exposed cysteines that can provide a significant oxidative aggregation (Kaplan et al., 1997), making it a poor choice for tagging oligomeric target proteins (Malhotra, 2009).

As occurs with MBP, GST can be coupled with other affinity strategies, for instance, the His_6_ tag, to improve the protein purification (Scheich et al., 2003; Hayashi and Kojima, 2008; Hu et al., 2008). GST expression vectors like the pGEX (Hakes and Dixon, 1992) or pCold-GST (Hayashi and Kojima, 2008) usually contain a protease recognition site between the fusion tag coding gene and the target protein coding gene for GST tag’s removal after or during protein purification.

GST has also been applied as a fusion partner in other expression systems apart from the *E. coli* such as yeast (Mitchell et al., 1993), insect cells (Beekman et al., 1994), and mammalian cells (Rudert et al., 1996). This fusion partner has shown to be useful for protein labeling (Ron and Dressler, 1992; Viljanen et al., 2008), antibody production (Aatsinki and Rajaniemi, 2005), and vaccine development (Mctigue et al., 1995).

In addition to these commonly used fusion partners, new solubility enhancer tags are constantly emerging in literature (see the corresponding references in **Table 1**), as for instance, the *Fh8* tag [see The Novel Fh8 Fusion System (Hitag®)], *HaloTag*, which uses a modified haloalkane dehalogenase protein that improves protein solubility and can bind to several synthetic ligands, the monomeric mutant of Orc protein of the bacteriophage T7 (*Mocr*), the *E. coli* protein *Skp*, stress-responsive proteins *RpoA*, *SlyD*, *Tsf, RpoS*, part of the domain I of IF2 (*expressivity tag*), the *E. coli* secreted protein A (*EspA*), and the *SNUT* tag, which is a protein derived from a portion of the bacterial transpeptidase sortase A of *Staphylococcus aureus*.

#### Affinity purification handles

Affinity fusion partners have widely contributed for the development of recombinant protein production studies in basic research and in HTP structural biology (Waugh, 2011) by simplifying protein purification procedures, and allowing for protein detection, and characterization (Butt et al., 2005; Malhotra, 2009; Young et al., 2012).

Affinity purification handles can be divided into two groups: (1) peptides or proteins that bind a small ligand immobilized on a solid support, as for instance, the His_6_ tag and nickel affinity resins, and (2) tags that bind to an immobilized molecule such as antibodies (Arnau et al., 2006).

The purification of a target protein using an affinity handle offers several *advantages* over the conventional chromatographic methodologies, namely:

(i) The target protein never interacts directly with the chromatographic resin (Waugh, 2005);

(ii) Target proteins can be easily obtained pure after a single-step purification (Terpe, 2003);

(iii) Affinity purification offers a variety of strategies to bind the target protein on an affinity matrix (Malhotra, 2009);

(iv) Affinity tags are an economically favorable and time-saving strategy, and they allow different proteins to be purified using a common method in contrast to highly customized procedures used in conventional chromatographic purification (Arnau et al., 2006).

An affinity tag is often chosen taking into account the purification costs: different affinity media and elution principles present different expenses during the operation process and should therefore be carefully selected at the beginning of the cloning strategy. The buffer requirements are also essential for the designing of an efficient purification strategy (Malhotra, 2009). In addition, the choice of an affinity can also rely on the size: small tags are useful for protein detection and antibody production, as they are not immunogenic as large tags (Terpe, 2003).

*Tandem affinity purification (TAP) *or* dual-tagging *strategies are now commonly used in recombinant protein production: they offer a highly specific isolation of target proteins with minimal background and under mild conditions, and they are very useful in the study of protein interactions, allowing the separation of different mixed protein complexes (Arnau et al., 2006; Li, 2010).

**Table 2** lists some of the common and novel purification tags used in recombinant protein production.

**Table 2:** Affinity purification tags [adapted from Esposito and Chatterjee (2006), Malhotra (2009)].

Tag	Protein	Size (aa)	Affinity matrix	Elution	Reference
His_6_	Hexahistidine tag	6–10	Immobilized metal ion – Ni, Co, Cu, Zn	Competition with imidazole	Gaberc-Porekar and Menart (2001)
Fh8	*Fasciola hepatica* 8-kDa antigen	69	Hydrophobic (calcium dependent interaction)	Ca^2+^-chelating agents such as EDTA or pH manipulation	Costa (2013), Costa et al. (2013b)
GST	Glutathione-*S*-transferase	211	Glutathione	Competition with free glutathione	Smith and Johnson (1988)
MBP	Maltose-binding protein	396	Amylose	Competition with maltose	di Guan et al. (1988), Pryor and Leiting (1997)
FLAG	FLAg tag peptide	8	Anti-FLAG antibody octapeptide when using anti-FLAG M2 antibody	Competition with FLAG	Einhauer and Jungbauer (2001)
Strep-II	Streptavidin binding peptide	8	Streptavidin	Competition with biotin and derivatives	Schmidt and Skerra (1994)
CBP	Calmodulin-binding protein	26	Immobilized calmodulin	Ca^2+^-chelating agents	Vaillancourt et al. (2000)
HaloTag	Mutated dehalogenase	~300	Chloroalkane	Covalent binding and proteolytic release of target protein	Ohana et al. (2009)
Protein A	Staphylococcal Protein A	280	Immobilized IgG	pH manipulation (acidic)	Stahl and Nygren (1997)
IMPACT (CBD)	Intein mediated purification with the chitin-binding domain	51	Chitin	Intein self-cleavage induction with dithiothreitol, β-mercaptoethanol or cysteine	Chong et al. (1997), Sheibani (1999)
CBM	Cellulose-binding module	^*^	Cellulose	Urea and guanidine–HCl or ethylene glycol	Tomme et al. (1998), Ramos et al. (2010), Ramos et al. (2013)
Dock	Dockerin domain of *Clostridium josui*	22	Cohesin – Cellulose	Ca^2+^-chelating agents	Kamezaki et al. (2010)
Tamavidin	fungal avidin-like protein	~140	Biotin	Free biotin in excess when using the Tamavidin 2-REV tag	Takakura et al. (2010, 2013)

* Several sizes, from 4 to 20 kDa.

The *polyhistidine affinity *tag or* His *tag consists of a variable number of consecutive histidine residues (usually six) that coordinate, via the histidine imidazole ring, transition metal ions such as Ni^2^^+^ or Co^2^^+^ immobilized on beads or a resin for IMAC (Gaberc-Porekar and Menart, 2001; Terpe, 2003; Kimple and Sondek, 2004; Malhotra, 2009). Commonly used IMAC resins such as nitrilotriacetic acid agarose (Ni–NTA, from Qiagen), or carboxymethylasparte agarose (Talon, from ClonTech) have a high binding capacity, and can be used for purification of fusion proteins directly from crude cell lysates (Terpe, 2003; Kimple and Sondek, 2004; Li, 2010).

The His tag is one of the most widely used purification tags, and it offers several advantages (Kimple and Sondek, 2004; Li, 2010):

(i) Its small size and charge rarely interferes with protein function and structure;

(ii) It can be used under native and denaturing conditions

(iii) Target proteins can be eluted under mild conditions by imidazole competition or low pH.

The His tag has been used in several HTP screenings, placed at the N- or C-terminal end, or even in the middle of the fusion protein (Cabrita et al., 2006; Hammarstrom, 2006; Marblestone et al., 2006; Bird, 2011), and it is also an useful tool in protein crystallization as well as protein detection (Carson et al., 2003; Kimple and Sondek, 2004).

Taking into account the mechanism of protein interaction with the immobilized ions, careful should be taken in IMAC to avoid strong reducing and chelating agents in any of the buffers (as for instance, EDTA), as they will reduce or strip the immobilized metal ions (Carson et al., 2003; Kimple and Sondek, 2004; Li, 2010).

*Epitope *tags are short sequences of amino acids that serve as the antigen region to which the antibody binds, being suitable for several immunoapplications. These include affinity chromatography on immobilized monoclonal antibodies, and protein trafficking *in vitro* or in cell cultures (Kimple and Sondek, 2004; Young et al., 2012). Epitope tagging engages an expensive purification that often limits its wide application.

The following partners are often used as epitope tags: the FLAG tag (Einhauer and Jungbauer, 2001), the hemaglutinin, and the c-Myc (Fritze and Anderson, 2000). Their short sequences rarely interfere with structure or function of target proteins, and are very specific for their respective primary antibodies (Kimple and Sondek, 2004; Malhotra, 2009). The *FLAG* tag is a short hydrophilic eight amino-acid peptide, and it was the first tag to be used in the epitope context. This tag works either for protein detection or purification (Hopp et al., 1988; Knappik and Pluckthun, 1994), and it has an intrinsic enterokinase cleavage site at its C-terminus end, allowing its complete removal from the target protein (Einhauer and Jungbauer, 2001; Young et al., 2012).

*Strep II *tag is a short tag of only eight amino acid residues that possesses a strong and specific binding to streptavidin via its biotin pocket (Schmidt and Skerra, 1994). This affinity partner can be fused at both N- or C-terminal ends, or within the target protein. Strep II-fused proteins elute from streptavidin columns with biotin derivates under gentle conditions (Terpe, 2003; Li, 2010).

The *CBP *tag is a calmodulin-binding peptide derived from the C-terminus of skeletal muscle myosin light chain kinase, and it has been used as an N- or C-terminal affinity tag of target protein purification on a calmodulin immobilized matrix (Terpe, 2003; Malhotra, 2009). The CBP interaction with calmodulin is calcium-dependent, and hence, the addition of calcium-chelating allows the single step elution of target proteins under gentle conditions (Terpe, 2003; Malhotra, 2009; Li, 2010). This tag is an affinity system highly specific for protein purification in *E. coli* but not in eukaryotic systems, as *E. coli* does not contain endogenous proteins that interact with calmodulin (Terpe, 2003; Malhotra, 2009).

In addition to the above-mentioned affinity tags, new affinity purification strategies are now described in literature for protein isolation and detection (see the corresponding references in **Table 2**) such as the *Fh8* tag [see The Novel Fh8 Fusion System (Hitag®)], cellulose-binding domains I, II, and III (*CBD*), the *HaloTag*, the dockerin domain *Dock* tag, and the avidin-like protein, *Tamavidin* tag.

#### Tag removal

The removal of the fusion partner from the final protein is often necessary because the tag can potentially interfere with the proper structure and functioning of the target protein (Waugh, 2005; Malhotra, 2009; Young et al., 2012).

Fusion partners are removed from their target proteins either by *enzymatic cleavage*, in which site specific proteases are used under mild conditions, or by *chemical cleavage*, like for instance formic acid (Ramos et al., 2010, 2013), that offers a less expensive tag removal but it is also less specific compared to the enzymatic strategy, besides presenting harsh conditions that can affect the target protein stability and solubility (Malhotra, 2009; Li, 2011). Fusion partners can also be cleaved from the target protein using an *in vivo* cleavage strategy, in which a controlled intracellular processing (CIP) is applied as follows: the fusion protein and protease are produced from separate compatible expression vectors that can be regulated independently of one another. The protease cleaves the fusion protein *in vivo*, offering the advantage of not compromising the target protein’s purity level or its production yields like often occurs in *in vitro* cleavage strategies (Kapust and Waugh, 2000).

The efficiency of the *enzymatic* removal of fusion proteins may vary in an unpredicted manner with different proteins (Li, 2011; Vergis and Wiener, 2011; Young et al., 2012), and it often requires the optimization of cleavage conditions through a trial-and-error process (Malhotra, 2009). Two types of proteases can be used for tag removal (reviewed in Waugh, 2011):

(i) *Endoproteases*: they are divided into *serine proteases* such as the activated blood coagulation factor X (factor Xa), enterokinase, and α-thrombin, and *viral proteases* like the tobacco etch virus (TEV), and the human rhinovirus 3C protease (**Table 3**). In spite of recognizing a similar number of substrate amino acid residues, viral proteases have usually more stringent sequence specificity than serine proteases, presenting also slower rates than the latter. Endoproteases are useful tools for the removal of N-terminal fusion tags, since they cleave close to the C-terminus end of their recognition sites thus leaving the target protein with its native N-terminal sequence.

**Table 3:** Common endoproteases for tag removal [adapted from Malhotra (2009)].

Protease	Source	Cleavage site	Reference
TEV	Tobacco etch virus protease	ENLYFQ/G	Parks et al. (1994), Kapust et al. (2001)
EntK	Enterokinase	DDDDK/	Choi et al. (2001)
Xa	Factor Xa	IEGR/	Jenny et al. (2003)
Thr	Thrombin	LVPR/GS	Jenny et al. (2003)
PreScission	Genetically engineered derivative of human rhinovirus 3C protease	LEVLFQ/GP	Cordingley et al. (1990), GE Healthcare (2010)
SUMO protease	Catalytic core of Ulp1	Recognizes SUMO tertiary structure and cleaves at the C-terminal end of the conserved Gly–Gly sequence in SUMO	Malakhov et al. (2004), Butt et al. (2005), Marblestone et al. (2006)

(ii) *Exoproteases*: they are often used together with endoproteases mainly for the removal of C-terminal fusion tags. The available exoproteases include metallocarboxypeptidases, and aminopeptidases.

The removal of a fusion tag is usually accomplished by two purification steps, as follows: after the initial affinity purification step (e.g., via a histidine tag located at the N-terminal of the fusion protein), the purified fusion protein is mixed in solution with the endoprotease (e.g., a his-tagged protease) to cleave off the tag. The cleaved target protein is recovered in the flow-through sample after a second affinity purification step, in which the cleaved fusion tag and the added protease are collected in the eluted sample.

In spite of widely employed, the removal of fusion partners has always been the Achilles’ heel of affinity tagging, presenting several *difficulties* such as:

(i) Unspecific cleavage due to the recognition of a linear amino acid sequence (except for SUMO protease);

(ii) Inefficient processing due to steric hindrance or the presence of unfavorable residues around the cleavage site (Li, 2011; Waugh, 2011). The inclusion of extra amino acid residues (a spacer or linker) between the cleavage site and target protein (Esposito and Chatterjee, 2006; Malhotra, 2009) can alleviate this problem;

(iii) Low protein yields after tag removal, and failure in recover active, structurally organized target proteins due to protein precipitation and aggregation (Butt et al., 2005; Waugh, 2011);

(iv) High costs of proteases and tedious optimization of cleavage conditions (Smyth et al., 2003).

Independently of the cleavage type, additional chromatographic steps are often required to purify the target protein from the cleavage mixture. Although conventional affinity technologies have greatly simplified recombinant protein production, resins, and buffers are still too expensive. Hence, the tag removal adds another layer of complexity and expense to the recombinant protein production process (Mee et al., 2008; Li, 2011).

*Self-cleaving tags* are a special group of fusion tags that possess inducible proteolytic activity, therefore being considered an attractive alternative to the existent affinity strategies for simple and costless protein purification and tag removal (Chong et al., 1997; Li, 2011).

The protein splicing is a process in which the intervening sequence (intein) removes itself and binds the flaking residues (exteins) to produce two independent protein products (Perler et al., 1994). Self-cleaving tags undergo specific cleavage upon being triggered by low molecular weight compounds or upon a change of conformation. The available technologies include inteins, the *S. aureus* sortase A, the N-terminal protease (N^pro^), the *Neisseria meningitides* iron-regulated protein FrpC, and the cysteine protease domain secreted by *Vibrio cholerae*, all of them reviewed in Li (2011).

## THE NOVEL Fh8 FUSION SYSTEM (Hitag®)

*Fh8* (GenBank ID: AF213970.1) is one of the promising new fusion technologies, advancing the existing tags by acting simultaneously as an effective solubility enhancer partner (Costa et al., 2013a) and robust purification handle (Costa et al., 2013b). Actually, the Fh8 is one of the few existent fusion tags to offer this combined feature of enhancing protein solubility and purification, and its low molecular weight (8 kDa) is also a great advantage over other large fusion partners for recombinant protein production in *E. coli *(Costa, 2013).

The Fh8 is a small antigen (8 kDa) excreted-secreted by the parasite *F. hepatica* in the early stages of infection (Silva et al., 2004). This protein is located on the surface of the parasite, and it was suggested as a useful tool for the diagnosis, vaccine, and drug development against *F. hepatica* infections (Silva et al., 2004). The use of recombinant Fh8 produced in *E. coli* led to the development of a novel, rapid, and simple immunodetection of *F. hepatica* infections (Silva et al., 2004). Moreover, when produced recombinantly in *E. coli*, the Fh8 revealed to be a highly soluble and unusual thermal stable protein (keeping secondary structure integrity up to 74°C; Silva et al., 2004; Fraga et al., 2010).

The Fh8 has high homology with 8-kDa calcium-binding proteins (CaBPs) of *Schistosoma mansoni* (Sm8; Ram et al., 1989), of *Clonorchis sinensis* (Ch8), and of *S. japonicum* (Sj8; Lv et al., 2009), and it belongs to the calmodulin-like EF-hand CaBP family (Fraga et al., 2010).

CaBPs are structurally organized by EF-hand motifs, which are helix–loop–helix structures that participate in Ca^2^^+^ coordination (Bhattacharya et al., 2004; Zhou et al., 2006; Chazin, 2011). Upon calcium binding, *Ca^2+^sensor proteins*, like calmodulin (Nelson and Chazin, 1998; Chin and Means, 2000) and troponin C (Nelson and Chazin, 1998), translate the physiological changes in calcium levels by undergoing a conformational change. This then allows the binding of other proteins downstream the process. In EF-hand proteins, the open of the EF-hand structure exposes a hydrophobic surface, which binds the target sequence (Lewit-Bentley and Rety, 2000; Bhattacharya et al., 2004). *Ca^2+^ buffer proteins*, such as calbindin D_9k_ and parvalbumin (Schwaller, 2010), are involved in calcium signal modulation, undergoing minimal conformational changes upon calcium binding.

The Fh8 presents two EF-hand motifs, and it was characterized as a *Ca^2+^sensor protein*: when calcium binds, the Fh8 switches from a closed (apo-state) to an open (calcium-loaded state) conformation due to the reorientation of the four helices, exposing a large hydrophobic region that acts as a target-binding surface (Fraga et al., 2010).

Previous studies for the prediction of the Fh8 three-dimensional structure (unpublished data) showed that almost all the Fh8’s amino acid sequence is involved or affected by the calcium-binding, with the exception of small residue sequences in the N-terminal (11 amino acid residues) and C-terminal (six amino acid residues). Considering that the N-terminal of a protein is very important for its half-life, the first N-terminal 11 residues of Fh8 were named the “H sequence” and were initially suggested to play a key role in the stability and production of the entire Fh8 protein. This H sequence could also be critical for the immunological response of the Fh8 antigen.

Taking into account the Fh8 high solubility and stability when expressed in *E. coli* together with its calcium-binding properties, and given the potential importance of the H sequence, both Fh8 and H peptides were suggested to function as fusion tags for protein production and solubility in *E. coli*, protein purification, and antibody production.

The application of both Fh8 (8 kDa) and H (1 kDa) peptides as fusion tags for protein overproduction in *E. coli* was first reported by Conceição and co-workers, using the following recombinant proteins: a 12-kDa surface protein of *Cryptosporidium parvum* (CP12), the interleukin-5 of human origin (IL-5), and an oocyst wall protein of *Toxoplasma gondii* (TgOWP; Conceição et al., 2010). This initial study showed that both Fh8 and H peptides have indeed a positive effect on the *E. coli* production levels of all target proteins, reaching values three- to 16-fold higher than those obtained with non-fused target proteins.

**FIGURE 2:**
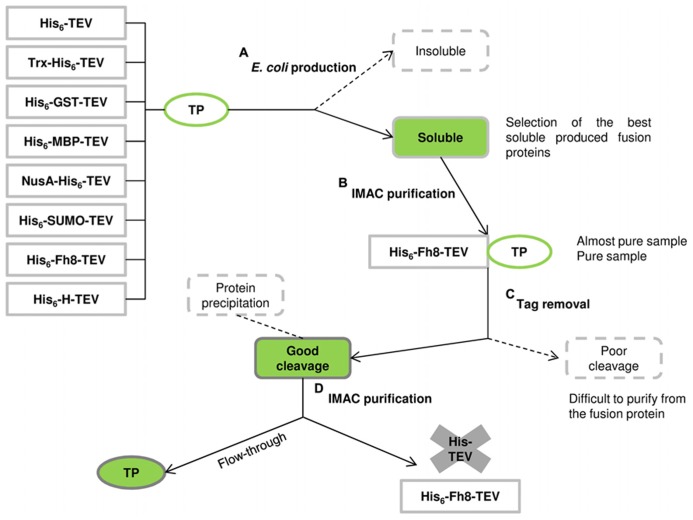
**Schematic pathway from protein production to purification using the solubility tags and the hexahistidine (His_6_) affinity tag of the comparison conducted by Costa et al. (2013a; adapted from Esposito and Chatterjee, 2006).** **(A)** Eight tagged versions of the TP were expressed in *E. coli*: some fusions can end-up in the insoluble fraction whereas others remain in the soluble fraction. **(B)** Soluble fusion proteins are then purified by immobilized metal affinity chromatography (IMAC) using the His_6_ tag and the fusion tags are removed from the TP by protease cleavage. **(C)** Some fusions will not cleave efficiently, resulting in a mixture of cleaved and uncleaved proteins that are difficult to separate. **(D)** Other fusions will cleave efficiently, and the TP remain in solution, being collected in the flow-through sample of a second IMAC purification step (as occurred with the Fh8 tag). Despite a successful protease cleavage, some TPs can become insoluble after tag removal leading to protein precipitation.

The Fh8 and H fusion tags were then studied as solubility enhancer tags, and their performance was compared with other commonly used fusion tags available in the Protein Expression and Purification Core Facility of the European Molecular Biology Laboratory (Costa, 2013; Costa et al., 2013a). **Figure 2** illustrates the schematic pathway from protein production to purification with the studied solubility tags (His_6_ tag, GST, MBP, NusA, Trx, SUMO, H, and Fh8). Here, the selected target proteins included the 12-kDa surface protein of *C. parvum* (CP12), the lectin frutalin from the *Artocarpus incisa *plant (FTL; Oliveira et al., 2008, 2009a,b, 2011), and four proteins from the yeast *S. cerevisiae*: reduced viability upon starvation protein 167 (RVS167), phospholipase D1 (SPO14), and serine/threonine-protein kinases 1 and 2 (YPK1 and YPK2). These target proteins were all known as difficult-to-express in *E. coli*, and presented different molecular weights, locations, and functions. The evaluation of their solubility and consequent effect of each fusion tag was performed after nickel affinity purification and upon tag removal in 10-mL cultures and in 500-mL cultures.

This comparison study showed that the Fh8 fusion partner stands among the well-described best fusion partners, MBP, NusA, and Trx, for soluble protein production. For the proteins tested, both GST and H fusion tags did not improve target protein solubility in *E. coli*.

The novel Fh8 fusion partner is thus an excellent candidate for testing production and solubility next to the other well-known fusion tags. Its low molecular weight and its solubility enhancing effect make Fh8 an advantageous option compared to larger fusion tags for soluble protein production in *E. coli*.

Apart from its solubility enhancer effect, the Fh8 was also explored by Costa (2013), Costa et al. (2013b) as a purification handle via its calcium-binding behavior combined with HIC. Two different model proteins were used within this study: green fluorescent protein (GFP) and superoxide dismutase (SOD), and the Fh8-HIC performance was also compared to the one of His tag technology (via IMAC).

**FIGURE 3:**
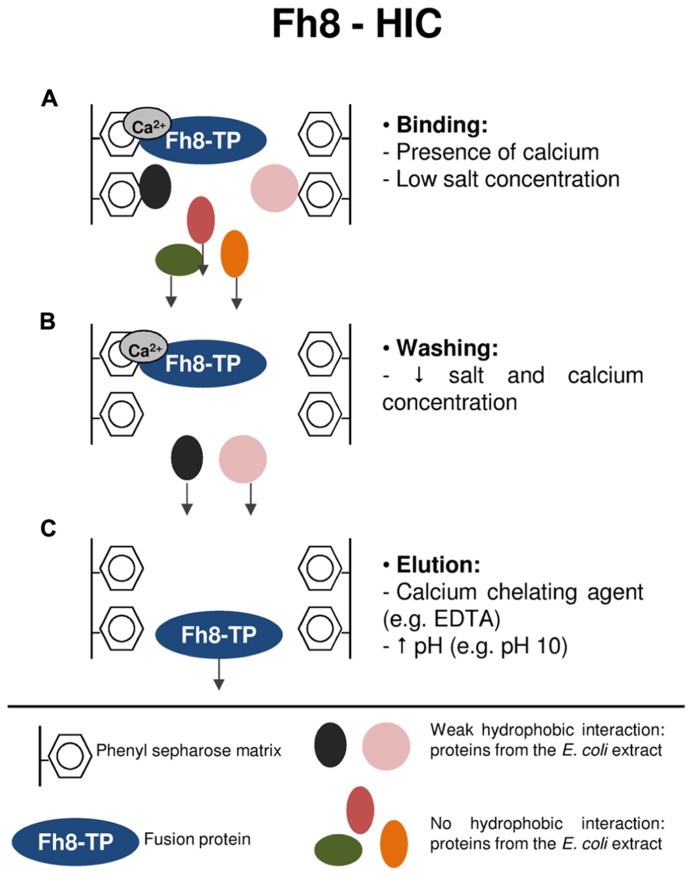
**Protein purification strategy using the Fh8-HIC methodology.** **(A)** Binding step: the Fh8-fused protein interacts with the hydrophobic matrix in the presence of calcium and at low salt concentration. This initial binding condition decreases the unspecific binding of other proteins from the *E. coli* extracts, which leave the column in the flow-through sample. **(B)** Washing step: by lowering the salts and calcium concentration, weakly interacting contaminant proteins are washed-out, and the Fh8-fused protein remains attached to the hydrophobic matrix. **(C)** Elution step: a calcium chelating agent, as for instance EDTA, will interfere in the calcium-dependent binding of the Fh8-fused protein, resulting in its elution from the hydrophobic matrix. The Fh8-fused protein can also be eluted by an alternative method: increasing the pH of the elution buffer to 10. This rise in the pH will promote a net charge around the fusion protein, which destabilizes the hydrophobic interactions and results in the elution of the fusion protein.

**Figure 3** resumes the purification mechanism of target proteins using the Fh8-HIC strategy. As previously mentioned, the Fh8 is a Ca^2^^+^-sensor protein that opens its structure upon calcium accommodation. The opening of the Fh8’s structure exposes a large hydrophobic surface that becomes available for interaction with its targets (Fraga et al., 2010). In this study, the Fh8 tag and Fh8-fused proteins presented a calcium-dependent interaction with a hydrophobic resin, and, as reported for other calcium-binding proteins (Rozanas, 1998; Shimizu et al., 2003), this interaction was still occurring even with low salt concentration in the mobile phase. The low salt concentration decreases the unspecific binding of other proteins from the *E. coli* extracts, thus promoting selectivity toward the purification of the fusion protein of interest (Costa et al., 2013b). Moreover, it was also shown that, as a calcium-binding protein, the Fh8 tag and Fh8-fused proteins can be eluted by using a calcium chelating agent, such as EDTA. One can also use for elution a mobile phase with an increased pH (e.g., pH 10), which creates a net charge that destabilizes hydrophobic interactions. This elution strategy allows a single-step and rapid elution of all bound proteins (Costa et al., 2013b).

The Fh8-HIC methodology presented also the advantage of being compatible with the IMAC technique, thus, allowing a dual protein purification strategy that can be used sequentially, complementing each other, to obtain an active and more purified protein when desired. In addition, the use of two consecutive purification steps and the distinct nature of HIC and IMAC methodologies is known to help for the efficient removal of contaminating proteins (McCluskey et al., 2007).

Regarding the H tag, it did not function as a solubility enhancer tag, but it improved the production levels of target proteins in *E. coli* similarly to the Fh8 tag (Costa et al., 2013a). Taking that into account, the H tag was further explored for the recombinant production of antigens of interest in *E. coli*, and their subsequent immunization and polyclonal antibody production.

**FIGURE 4:**
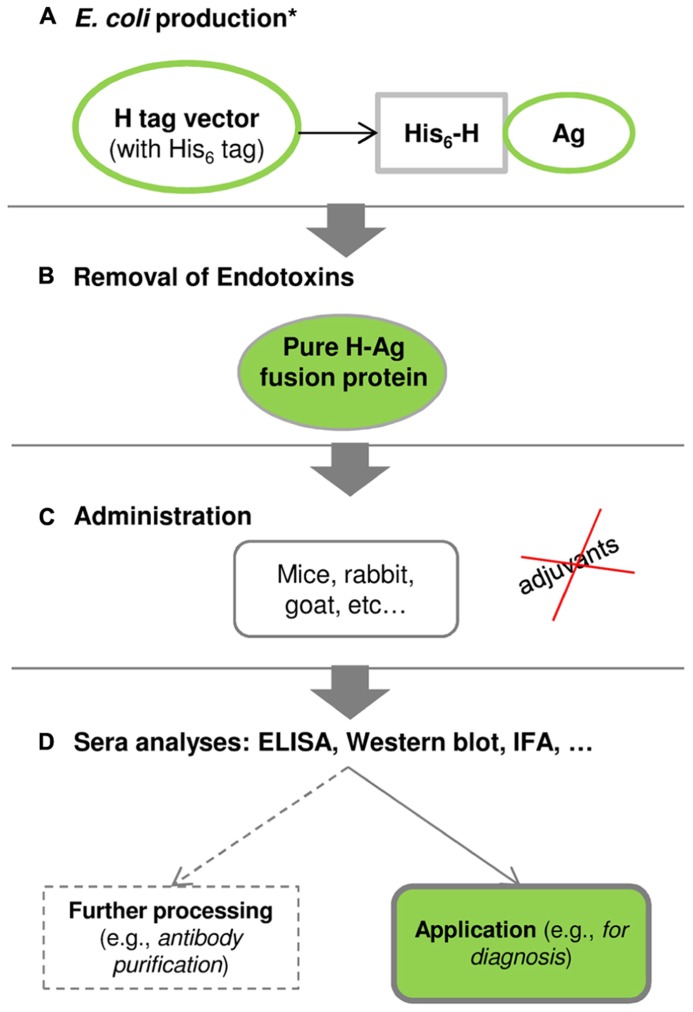
**The schematic pathway from gene to antibody using the H fusion tag (Costa et al., 2013c).** **(A)** Production of H-fused antigens in *E. coli*: *the antigen-codifying gene is inserted into a H-tag expression vector, and protein production and purification are optimized following the conditions presented in **Figure 1**. **(B)** *E. coli* endotoxins can be removed using a commercial endotoxin-removal kit or by hydrophobic interaction chromatography. **(C)** Purified H-fused antigens can be administrated into mice, rabbits, goats, among others, and this procedure is conducted without adjuvants. **(D)** The produced sera are analyzed by enzyme-linked immunosorbent assay (ELISA), Western blot, immunofluorescence assay (IFA), among others, to validate the specificity and practical application of polyclonal antibodies. Further processing may be required in order to obtain highly purified polyclonal antibodies.

The major novelty of the H tag relies on its small size (1 kDa) combined with the adjuvant-free immunization of antigens (Conceição et al., 2011; Costa, 2013; Costa et al., 2013c). **Figure 4** shows the schematic pathway of using the H fusion tag from gene to antibody.

Costa et al. (2013c) showed a successful case study with the CP12 antigen, which has a low molecular weight that can hinder the production of polyclonal antibodies. The HCP12 fusion antigen elicited an earlier immune response and higher (approximately 2-fold) polyclonal antibody titers than the non-fused CP12 (Conceição et al., 2011; Costa et al., 2013c). This application study demonstrated that the H partner improves the specific polyclonal antibody production against the CP12 antigen without using adjuvants, and the resulting polyclonal antibodies can be used as a diagnostic tool for immunodetection of *C. parvum* infections in humans or animals (Costa et al., 2013c).

Apart from CP12, several H-fused antigens have already been produced in *E. coli* (Conceição et al., 2010) and immunized in mice and rabbits, such as, the human interleukin-5 (IL-5), the cyst wall protein-1 from *T. gondii* (TgOWP), the cyst wall protein from *Giardia lamblia* cysts (CWG), the β-giardin cytoskeletal protein of the ventral disk from the *G. lamblia* trophozoite (βG), the cyst wall specific-glycoprotein Jacob from *Entamoeba histolytica* (Ent), and the falcipain-1 trophozoite cysteine proteinase from *Plasmodium falciparum* (Pfsp), among others (Conceição et al., 2011).

## CONCLUSIONS AND FUTURE TRENDS

The growing demand for effective health and environmental biotechnology resources has advancing the design of different strategies for the successful protein production in *E. coli*. Its benefits of cost and ease of use and scale make *E. coli* one of the most widely used host systems for recombinant protein production, but one must be aware that success is not always guaranteed in this prokaryotic host system, mainly when working with recombinant proteins of human origin.

This review highlighted several key factors that contribute to the soluble protein production and purification in *E. coli*, including the use of different mutated host strains, co-production of chaperones and foldases and testing different cultivation conditions, with a main focus in the gene fusion technology.

The use of fusion partners was an important turning point for the *E. coli* host system: fusion tags promote or increase protein solubility, help on protein purification and can also be used to increase protein’s immunogenicity. Traditional fusion systems like MBP, GST, NusA, or Trx have constantly been challenged and complemented by novel fusion solutions such as the SUMO tag (Butt et al., 2005; Marblestone et al., 2006), the HaloTag (Ohana et al., 2009), the SNUT tag (Caswell et al., 2010), and the expressivity tag (Hansted et al., 2011), among others.

More recently, a novel and unique fusion system for simple and inexpensive soluble protein overproduction and purification in *E. coli* was developed and studied: the Fh8 tag (Costa, 2013).

The Fh8 is ranked among the best solubility enhancer tags as Trx, MBP, or NusA (Costa et al., 2013a), and it offers a specific and simple purification of the target proteins by using its natural calcium-binding properties and mild conditions for HIC (Costa et al., 2013b). The Fh8 fusion partner is one of the few existing tags to promote simultaneously target protein solubility directly into the *E. coli* cytoplasm and a simple and cost-effective protein purification.

The novel Fh8 fusion system overcomes several issues related with recombinant protein production in *E. coli*: by using a straightforward methodology, this novel system increases protein production levels, promotes protein solubility and low cost purification, and helps for protein immunogenicity, in which the H tag facilitates a simple, rapid, and adjuvant-free production from gene to antibody (Costa et al., 2013c). This novel fusion system offers the great advantage of combining these four abilities into the two lowest molecular weight fusion partners described so far. Hence, the Fh8 fusion system appears as a valuable tool for the efficient and economical recombinant protein production in *E. coli*.

While this review applies to the use of Fh8 and H tags for recombinant protein production in bacterial host systems, it is hoped that the novel fusion system presented here will apply to other hosts, as for instance, eukaryotes and mammalian cells and thus, this must be investigated.

Despite being widely employed to improve soluble protein production in *E. coli*, fusion tags are not yet well comprehended as suggested by the general lack in literature of studies regarding their mechanism of action. Therefore, efforts should be taken to disclose how fusion tags work while promoting such a positive effect in the protein production in *E. coli*. Perhaps, a wide systems biology analysis can help to reveal the different pathways that fusion tags undergo in *E. coli*, leading also to their organization into functional groups.

Taking into account the broad range of applications, the trend is that the number of available fusion tags will increase, and the understanding of their way of action will, undoubtedly, allow the development of tailored-made tools for protein production.

## AUTHOR CONTRIBUTIONS

Sofia Costa drafted the review, participated in the study design of the novel Fh8 and H fusion tags, and in most of its experimental work. André Almeida, António Castro, and Lucília Domingues participated in the study design of the novel Fh8 and fusion tags. Lucília Domingues conceived and helped to draft the review. All authors read and approved the final manuscript.

## Conflict of Interest Statement

The Fh8 tag utilization for the improvement of protein production in *E. coli* and the Htag utilization for the production of immunogens and corresponding polyclonal antibodies are covered by worldwide patents (WO 2010082097 and WO 2011071404, respectively), both licensed to Hitag Biotechnology, Lda. The authors Sofia Costa, André Almeida, and António Castro are co-owners of the patent and are associated with Hitag Biotechnology, Lda.
